# HIV-1/2 differentiation in a South African public laboratory

**DOI:** 10.4102/sajhivmed.v22i1.1185

**Published:** 2021-03-12

**Authors:** Rendani T. Mafuyeka, Lynne M. Webber, Piet Becker, Simnikiwe H. Mayaphi

**Affiliations:** 1 Department of Medical Virology, Faculty of Health Sciences, University of Pretoria, Pretoria, South Africa; 2 Department of Virology, Tshwane Academic division, National Health Laboratory, Pretoria, South Africa; 3 Research Office, Faculty of Health Sciences, University of Pretoria, Pretoria, South Africa

**Keywords:** HIV-1/2 differentiation, HIV-2 testing, HIV1/2 antibody cross reaction, HIV-2 in South Africa, HIV-2 PCR

## Abstract

**Background:**

The human immunodeficiency virus type-2 (HIV-2) prevalence in South Africa (SA) is unknown, however, sporadic cases have been reported. Human immunodeficiency virus -1 and 2 differentiation is not part of most South African public laboratories’ testing algorithm. Human immunodeficiency virus -2 diagnosis using serology assays may be complicated by HIV-1 and HIV-2 antibody cross-reactivity.

**Objectives:**

To determine the proportion of HIV-2 infections in specimens that tested HIV-1/2 positive at a public laboratory in Tshwane.

**Method:**

A total of 480 specimens that were previously tested with fourth generation ELISA platforms (Modular E170 [Roche, Switzerland] and Architect i2000 [Abbott, Germany]) were randomly selected. Human immunodeficiency virus -1 and 2 antibody differentiation testing was carried out using the Multispot HIV-1/2 rapid assay (Bio-Rad Laboratories, USA). An in-house nested HIV-2 PCR assay targeting the 5′-long terminal repeats (5′-LTR) region was evaluated and used as a confirmatory test.

**Results:**

The study tested 480 HIV-1/2 seropositive patients and their mean age was 36.7 years (range 3–82 years). Of the 480 patients, 292 (60.8%) were female, 182 (37.9%) were male and 6 (1.3%) were not specified. Human immunodeficiency virus differentiation results were as follows: 466 (97.1%) were positive for only HIV-1 antibodies, 11 (2.3%) [95%CI: (0.98%; 3.74%)] were positive for both HIV-1 and HIV-2 antibodies, 3 (0.6%) were negative for both antibodies and none were positive for only HIV-2 antibodies. Of the 11 specimens with both HIV-1 and HIV-2 antibodies, seven had sufficient volume for confirmatory testing and were all negative on the in-house HIV-2 PCR assay.

**Conclusion:**

The multispot HIV-1/2 rapid assay demonstrated cross-reactivity between HIV-1 and HIV-2 antibodies. Human immunodeficiency virus -2 infections were not detected.

## Introduction

Human immunodeficiency virus type 2 (HIV-2) belongs to the *Retroviridae* family, *Lentivirus* genus.^1^ It is most prevalent in West Africa.^2^ Most countries with historical ties with West Africa have also detected a significant proportion of HIV-2 infections.^3^ Although the HIV pandemic is mainly caused by HIV-1, HIV-2 is also an important cause of acquired immunodeficiency syndrome (AIDS).^4^ Both HIV-1 and HIV-2 have similar clinical manifestations, however, HIV-2 progresses much slower to AIDS.^5^

South African data on HIV-2 infections is limited. Sporadic cases have been reported in the past, which is an indication that HIV-2 may be circulating in South Africa (SA).^6,7^ In addition, SA has a large number of tourists and immigrants from the West African region and other parts of the world where HIV-2 infections have been reported,^8,9^ which may contribute to the spread of the virus in the country.

Routine diagnostic serology assays that are widely used in SA national public sector health laboratories do not include HIV-1 and HIV-2 differentiation.^10^ This may have implications for HIV management,^11^ as the treatment of HIV-2 differs from that of HIV-1. Human immunodeficiency virus-2 is intrinsically resistant to non-nucleoside reverse transcriptase inhibitors (NNRTIs) and fusion inhibitors. It is also only weakly suppressed by some protease inhibitors. Human immunodeficiency virus-2 has a lower genetic barrier compared with HIV-1 to nucleoside reverse transcriptase inhibitors (NRTIs) resistance.

Dolutegravir is effective against HIV-2, but strains with raltegravir mutations may have low levels of resistance.^12^

The virology diagnostic laboratory is currently using a fourth generation enzyme-linked immunosorbent assay (4th gen ELISA) for HIV-1/2 testing. The fourth generation ELISA can detect both HIV-1 and HIV-2 antibodies and p24 antigen, but it does not differentiate between the two types. The World Health Organization (WHO) HIV testing guidelines recommend HIV-2 differentiation with serological and/or molecular assays in settings where HIV-2 infections have been detected.^13^ Human immunodeficiency virus -2 molecular testing is an important confirmatory assay, owing to the high HIV-1 and HIV-2 antibody cross-reactivity with serological assays.^14^

### Aims

This study aimed to determine the proportion of HIV-2 infections in specimens that tested HIV-1/2 positive in a public laboratory in Tshwane.

### Objectives

- Human immunodeficiency virus -1 and -2 differentiation using the Multispot HIV-1/2 rapid test.
- Evaluation of the in-house HIV-2 nested polymerase chain reaction (PCR) assay.
- Confirmatory testing using the in-house HIV-2 PCR assay.

## Methods and materials

### Study design

This was a retrospective, cross-sectional study conducted between February 2013 and July 2016.

### Study setting

This study was conducted in the National Health Laboratory Services (NHLS), Tshwane Academic Division (TAD) Virology Laboratory at the University of Pretoria, Faculty of Health Sciences. The specimens used came from the public sector hospitals and clinics around Tshwane Metropolitan Municipality, submitted for routine HIV screening.

### Specimen selection

A total of 480 plasma and serum specimens that had previously tested positive for HIV-1/2 antibody, were randomly selected. The inclusion criteria were age of more than 24 months and HIV-1/2 antibody positive results on two different fourth generation ELISA platforms, the Modular E170 (Roche, Switzerland) and the Architect i2000 (Abbott, Germany).

### Statistics

The HIV-2 prevalence is unknown in SA. Based on the HIV-2 prevalence data from other parts of the world outside the West African region,^15,16,17^ a prevalence of 1% was assumed for SA. It was determined that a sample size of 459 tests would have a 0.99 probability to detect at least one HIV-2 positive specimen. The sample size calculation was done using software nQuery version 7.0.^18^

### HIV-1 and 2 antibody differentiation

The Multispot HIV-1/2 rapid assay (Bio-Rad Laboratories, USA) was used for HIV-1 and HIV-2 differentiation.

The procedure was performed according to the manufacturer’s instructions. Four known HIV-2 antibody positive specimens, from a laboratory in Portugal, that were previously tested using only the Western Blot assay, and 52 HIV-1 and HIV-2 antibody negative specimens that were previously tested using HIV-1/2 fourth generation ELISA, Modular E170 (Roche, Switzerland) were included to evaluate performance of the Multispot HIV-1/2 rapid assay.

### In-house HIV-2 nested polymerase chain reaction assay

Two sets of primers, targeting the conserved 5′ long terminal repeat (5′-LTR) region of HIV-2, were designed and numbered according to the HIV-2 ROD reference strain, accession number M15390.1 (Table 1). The in-house HIV-2 nested PCR assay was evaluated using the four known HIV-2 antibody positive specimens. These specimens were also processed at a private laboratory using a commercial real-time HIV-2 PCR assay (Genesig Standard kit) that targets the integrase (pol) gene region. The Genesig standard kit detects HIV-2 subtypes A and B only. The internal control was also included in the commercial real-time assay. Two known HIV-1 specimens with high viral load (132 000 copies/mL and 30 200 copies/mL) were included to determine the specificity of the in-house PCR primers. The in-house HIV-2 nested PCR assay was used to confirm specimens that were positive for both HIV-1 and HIV-2 antibodies on the Multispot HIV-1/2 rapid assay.

**TABLE 1:** Designed primers used for the in-house nested human immunodeficiency virus-2 polymerase chain reaction and sequencing.

Primers	Positions	Sequences	Length (number of bases)	Tm† (°C)§
MMFw-1 (outer)	11–27	5՛-CGGAGAGGCTGGCAGAT-3՛	17	62
MMRv-1 (outer)	566–583	5՛-AGTTTCTCGCGCCCATCT-3՛	18	59.9
MMFw-2 (inner)	72–94	5՛-GTGTTCCCTGCTAGACTCTCACC-3՛	23	66.3
MMRv-2 (inner)	392–409	5՛-GGAGCACTCCGTCGTGGT-3՛	18	64.5

† Tm, melting temperature;

§ °C, degree celsius.

### Nucleic acid extraction

Except for the initial centrifugation step to get a purified pellet, the extraction of total nucleic acids was performed manually using the QIAamp UltraSens Virus kit (Qiagen, Germany) according to the manufacturer’s instructions.

Centrifugation was optimised at 800 relative centrifugal force (RCF) for 3 min instead of the recommended 1200 RCF. Optimisation to a lower RCF produced an easily dissolvable pellet. Sample input volume for extraction was 500 µL, and lower input volume of 250 µL was used for samples with insufficient volume. The final eluate volume was 60 µL.

### Amplification, detection and analysis

A nested PCR was carried out using the Superscript III One-Step RT-PCR kit with platinum Taq deoxyribonucleic acid polymerase (5 U/µL) (Life technologies, USA) according to the manufacturer’s instructions. The total reaction volumes for both first and second round PCR were 50 µL. First round PCR contained 25 µL of 2x reaction mix (a buffer containing 0.4 mM of each dNTP, 2.4 mM MgSO4); 1 µL of each primer (outer forward and reverse) (10 µM); 1 µL of Taq enzyme (5 U/µL), 5 µL of template and 17 µL of nuclease-free H_2_O. The second round PCR reaction contained 25 µL of 2x reaction mix (a buffer containing 0.4 mM of each dNTP, 2.4 mM MgSO4); 1 µL of each primer (inner forward and reverse)(10 µM); 0.2 µL of Taq enzyme (5 U/µL), 0.5 µL of template and 22.3 µL of nuclease-free H_2_O.

The first round PCR conditions were as follows: cDNA synthesis for 1 cycle at 50 °C for 30 min; denaturation for 1 cycle at 94 °C for 2 min; amplification 40 cycles (denature at 94 °C for 30 s, annealing at 50 °C for 30 s, extension at 68 °C for 1 min); final extension for 1 cycle at 68 °C for 7 min and hold at 4 °C. The second round PCR conditions were as follows: denaturation for 1 cycle at 94 °C for 2 min; amplification 40 cycles (denature at 94 °C for 30 s, annealing at 50 °C for 30 s, extension at 68 °C for 30 s); final extension for 1 cycle at 68 °C for 7 min and hold at 4 °C. Polymerase chain reaction amplicons were visualised on a 1.5% agarose gel by ultraviolet (UV) illumination.

### HIV-2 sequencing

Polymerase chain reaction amplicons were sequenced to evaluate the in-house HIV-2 nested PCR, at Inqaba Biotechnical Industries (Pty) Ltd using the second round PCR primers (MMFw-2 and MMRv-2) (Table 1). The original PCR products were sent to Inqaba where they performed a clean-up procedure and Sanger sequencing. Sequence analysis comprised of sequence editing and alignment performed using BioEdit Sequence Alignment Editor Version 7.2.5 programme.^19^ Molecular Evolutionary Genetics Analysis (MEGA) version 6 programme,^20^ was used for constructing the phylogenetic tree using Neighbor-Joining statistical method (with 1000 bootstrap replicates).

### Ethical consideration

This study was approved by the research ethics committee, Faculty Health Sciences, University of Pretoria (ethics reference number: 11/2013). Permission to use the blood specimens and to retrieve demographics information from the laboratory information system (LIS) was obtained from Tshwane Academic Division of the NHLS. The information of each specimen was managed with strict confidentiality and each specimen was given a unique study number to ensure patient anonymity.

## Results

### HIV-1 and 2 antibodies differentiation results

**FIGURE 1:**
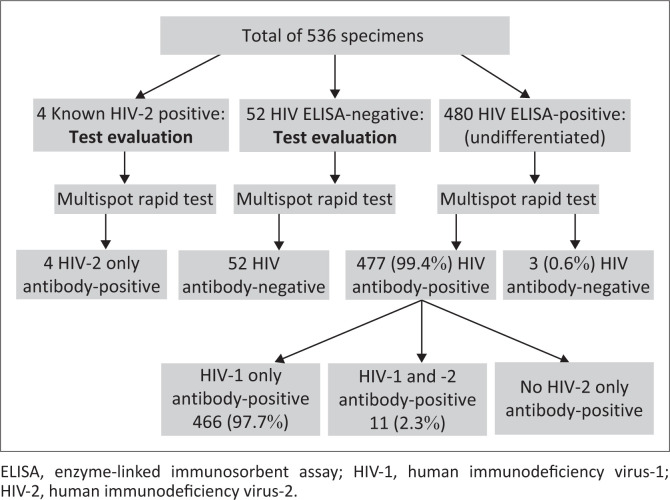
Algorithm of the specimens tested with Multispot HIV-1/2 rapid assay.

The study tested 480 HIV-1 and HIV-2 seropositive patients, and their mean age was 36.7 years (range: 3–82 years). Of the 480, 292 (60.8%) were female, 182 (37.9%) were male and 6 (1.3%) were not specified. All four known HIV-2 antibody positive specimens used to evaluate the Multispot HIV-1/2 rapid assay were positive for HIV-2 antibodies only. All 52 known HIV-1 and HIV-2 ELISA negative specimens were negative. Differentiation results of the 480 specimens were as follows: 466 (97.1%) were positive for only HIV-1 antibodies, 11 (2.3%) 95%CI: 0.98%; 3.74% were positive for both HIV-1 and HIV-2 antibodies, 3 (0.6%) were negative for both antibodies and none were positive for only HIV-2 antibodies (Figure 1).

### In-house HIV-2 nested polymerase chain reaction assay results

**FIGURE 2:**
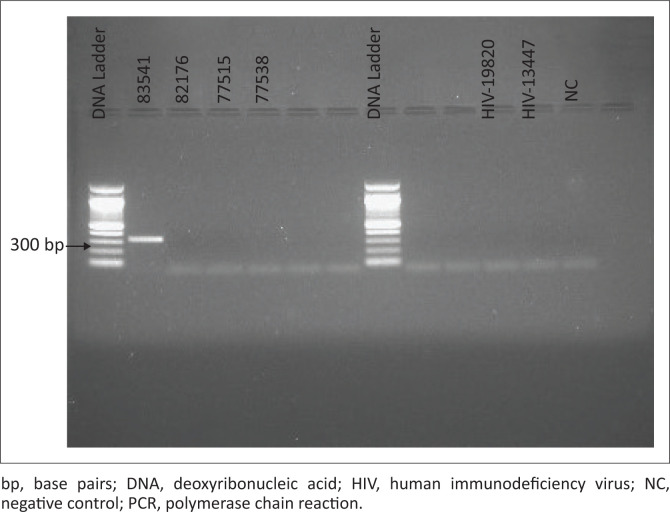
Gel Electrophoresis with a 100 bp DNA ladder. Specimens 83541, 82176, 77515 and 77538 are known HIV-2 antibody positive. Specimen 83541 tested positive for HIV-2 PCR (fragment size = 338 bp). HIV-1 specimens (9820 and 3447) with high viral load were undetectable.

Evaluation results of the in-house nested HIV-2 PCR, on four known HIV-2 antibody positive specimens, were comparable to a commercial real-time PCR, which was carried out in a private laboratory (Table 2). Two HIV-1 specimens, with high viral load, were also not detected by the in-house HIV-2 PCR (Figure 2). Of the 11 specimens, which were positive for both HIV-1 and HIV-2 antibodies, only seven had sufficient volume for further testing. All tested HIV-2 PCR negative on the in-house assay.

**TABLE 2:** HIV-2 polymerase chain reaction results of the four known HIV-2 antibody positive specimens.

Known HIV-2 specimens	In-house nested HIV-2 PCR	Genesig standard kit real time HIV-2 PCR (Private laboratory)	Internal Control for Real-Time PCR
83541	Positive	Positive	Positive
82176	Negative	Negative	Positive
77515	Negative	Negative	Positive
77538	Negative	Negative	Positive

HIV, human immunodeficiency virus; PCR, polymerase chain reaction.

### HIV-2 sequencing results

**FIGURE 3:**
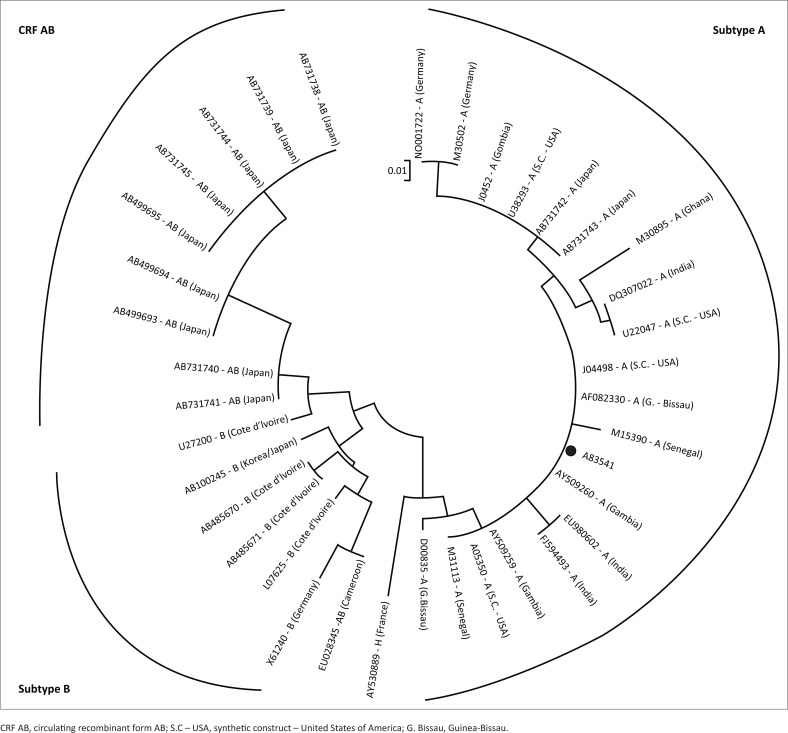
Phylogenetic tree showing the sequence of the known human immunodeficiency virus-2 sample that was positive on in-house polymerase chain reaction (labelled with a black dot). This sequence clustered with subtype A human immunodeficiency virus-2 isolates.

The PCR products of the confirmed HIV-2 subtype A/B specimen (83541) were sent for sequencing. Phylogenetic analysis revealed that this sequence clustered with subtype A of HIV-2 (Figure 3). Sequencing was not indicated for those specimens that were HIV-2 negative on the in-house PCR assay.

## Discussion

This study aimed to determine the proportion of HIV-2 infections in specimens that tested HIV-1/2 antibody positive at a virology diagnostic laboratory using the Multispot HIV-1/2 rapid assay for differentiation. The Multispot rapid test has been reported as satisfactory with regard to the detection of HIV-1 and HIV-2.^21,22,23,24,25,26^

Differentiation results showed HIV-1 and HIV-2 dual seropositivity in 11 (2.3%) of the specimens. Of the 11, seven specimens with sufficient volume for the in-house HIV-2 PCR tested negative. This may indicate HIV-1 and HIV-2 antibody cross-reactivity. These results were not surprising, as HIV-1 and HIV-2 antibody cross-reactivity have been observed in other studies.^24,27^ A study conducted in SA demonstrated high levels of false HIV-2 seropositivity, at 15%, 71.2% and 10.7%, using the SD Bioline HIV1/2 3.0 rapid test, the NewLAV Western blots, and Pepti-LAV1-2, respectively.^27^ This South African study demonstrated a high rate of antibody cross reactivity with negative HIV-2 molecular testing. Another study carried out in the United States of America, demonstrated antibody cross-reactivity rates of 46.5% and 11.4% using the Centres for Disease Control (CDC) HIV-1 Western blot (WB) interpretive criteria and the alternative more stringent HIV-1 WB interpretive criteria, respectively.^24^ The HIV-1 and HIV-2 antibody cross-reactivity rate was 2.3% in this study. Other previous studies that also used the Multispot assay demonstrated a cross-reactivity rate of 0.5%^26^ and 1.3%.^24^ These previous studies are further demonstrating that HIV-2 testing using serological assays may be complicated owing to high levels of antibody cross-reactivity and should be interpreted with caution. Highly sensitive and specific molecular confirmatory testing is always important for those specimens with dual HIV seropositivity^14^ and for research purposes.

The first two reported HIV-2 cases in SA were documented in 1988^6^ and 1993.^7^ These cases were both diagnosed and confirmed using antibody detection assays. Confirmation of the 1988 case was done using an immunofluorescence assay and that of the 1993 case was done using a Western blot assay. Possibility of HIV-1 and HIV-2 antibody cross reactivity in these two cases cannot be ruled out as no molecular testing was performed.

The in-house nested HIV-2 PCR had comparable results, on the four known HIV-2 antibody positive specimens, with the commercial assay (Genesig Standard kit) processed in a private laboratory (Table 2). Sequencing of the PCR products of the known HIV-2 positive specimen (83541) indicated HIV-2 subtype A (Figure 3). The other three HIV-2 seropositive specimens that failed to amplify using HIV-2-specific PCRs (the in-house and the commercial assays) may have reflected naturally low levels of HIV-2 virus or the effect of antiretroviral therapy exposure. There was no relevant clinical information provided on these known HIV-2 antibody positive specimens from the laboratory in Portugal, which could have explained these results. In addition, the in-house nested HIV-2 PCR assay did not detect HIV-1 in two specimens with high viral loads highlighting a lower likelihood of false positive results in individuals with HIV-1 infection. To further evaluate the sensitivity and specificity of the primers used for in-house nested PCR, more HIV-1 and HIV-2 samples need to be tested.

The negative HIV-2 PCR on specimens with dual HIV seropositivity may not completely rule out dual HIV infections, as there are many factors that may contribute to false-negative HIV-2 PCR. Such factors include naturally low viral load in HIV-2 infections because of the low replication rate.^28^ In a study by Drylewicz J et al., plasma HIV-2 viral load was undetectable in up to 85% of individuals positive for HIV-2 antibodies.^29^ Another possible explanation could be that HIV-1 suppresses HIV-2 replication in individuals with dual infection; hence, the HIV-2 antibodies and viral load remain very low or undetectable. The quality of specimens used in this study may have been compromised as well. Specimens were initially stored at 4 °C for more than 7 days prior to testing, which may have led to RNA degradation. Serum and plasma specimens used in this study may not be the best for HIV-2 detection as evidence suggests that HIV-2 replication is restricted *in vivo*.^30^ Peripheral blood mononuclear cells (PBMCs) may yield a better outcome with regard to HIV-2 detection.^30,31^

The limitations of this study include small sample size, quality and type of the specimens used as described here.

Low specimen volumes (250 µL – 500 µL) were used for extraction because of specimen insufficiency. Nucleic acid quantification and quality assessment were also not done. This may have led to false negative results. Polymerase chain reaction failure of the in-house PCR could not be excluded because of the lack of internal controls; however, the commercial HIV-2 PCR with comparable results and with internal controls, amplified. The developed in-house nested HIV-2 PCR needs further evaluation with more HIV-2 positive specimens, including other subtypes and addition of more HIV-1 only specimens. The commercial assay can only detect HIV-2 subtypes A and B.^32^ Detection of other HIV-2 subtypes (C–G) could not be evaluated.

## Conclusion

The Multispot HIV-1/2 rapid assay demonstrated some cross-reactivity between HIV-1 and HIV-2 antibodies. Human immunodeficiency virus-2 infections were not detected. Dual infections could not be excluded in this study. The in-house HIV-2 PCR assay needs further evaluation to determine its sensitivity and specificity. These results cannot be generalised because the sample size was too small. Further studies are needed to explore HIV-2 infections and treatment response in SA.

### Postscript

The Multispot HIV-1 and HIV-2 rapid assay was later discontinued in 2016 and was replaced by the Geenius HIV-1 and HIV-2 Supplemental Assay.^33^
